# Supplementary material for: Observed joint infection incidence following needle arthroscopy performed in operating and nonoperating room environments in client-owned dogs: A retrospective cohort study
Source: PLoS One. 2026 Feb 25;21(2):e0343878. doi: 10.1371/journal.pone.0343878 (PMC12935261; doi:10.1371/journal.pone.0343878)

PLOS ONE

A comparative analysis of infection rates associated with needle arthroscopy conducted in an operating room versus those performed under sedation, in a nonoperating room setting in canine joints: A retrospective study.

--Manuscript Draft--

**Manuscript Number:**	PONE-D-25-49231
**Article Type:**	Research Article
**Full Title:**	A comparative analysis of infection rates associated with needle arthroscopy conducted in an operating room versus those performed under sedation, in a nonoperating room setting in canine joints: A retrospective study.
**Short Title:**	Infection rates for outpatient needle arthroscopy in canine joints.
**Corresponding Author:**	Alessandra Chiaramonte, VMD<br>UTCVM: The University of Tennessee Knoxville College of Veterinary Medicine<br>Knoxville, TENNESSEE UNITED STATES OF AMERICA
**Keywords:**	Nanoscope<br>Needle arthroscopy<br>Canine joint arthroscopy<br>Outpatient needle arthroscopy
**Abstract:**	Objective<br><br>To determine infection rates for needle arthroscopy (NA) in client-owned dogs performed in an operating room (OR) versus in a nonoperating room setting (NOR) under heavy sedation. We hypothesized that there would be no difference in infection rate.<br><br>Study Design<br><br>Retrospective study of 75 dogs (89 individual joints) that underwent needle arthroscopy at one institution from May 2022 through January 2025 were compiled.<br><br>Results<br><br>Ten cases were excluded due to evidence of infection before arthroscopy for TPLO implant explant (n = 8), bite wound infection (n = 1), or surgical complications not related to the needle arthroscopy (n = 1). Forty-five cases were performed in an operating room under general anesthesia, while thirty cases were performed under heavy sedation in a non-operating room setting. Routine follow-up was performed. Descriptive statistics were reported since the infection rate was 0% for all OR and NOR. There was no difference in infection rate for OR needle arthroscopy or NOR needle arthroscopy.<br><br>Conclusion<br><br>Needle arthroscopy can be performed on an outpatient basis and without increased risk of a septic joint when performed with appropriate aseptic technique.
**Order of Authors:**	Alessandra Chiaramonte, VMD
	Cassio R. A. Ferrigno, DVM, MS, PhD
	Darryl L. Millis, DVM, MS, DACVS, DACVSMR
	Jessica W. Montoya, AAS, LVMT, CCRP, VTS-Surgery
**Additional Information:**	
**Question**	**Response**
**Financial Disclosure**	The author(s) received no specific funding for this work.

Powered by Editorial Manager® and ProduXion Manager® from Aries Systems Corporation

Enter a financial disclosure statement that describes the sources of funding for the work included in this submission. Review the submission guidelines for detailed requirements. View published research articles from *PLOS ONE* for specific examples.<br><br>This statement is required for submission and **will appear in the published article** if the submission is accepted. Please make sure it is accurate.<br><br>**Funded studies**<br>Enter a statement with the following details:<br>• Initials of the authors who received each award<br>• Grant numbers awarded to each author<br>• The full name of each funder<br>• URL of each funder website<br>• Did the sponsors or funders play any role in the study design, data collection and analysis, decision to publish, or preparation of the manuscript?<br><br>Did you receive funding for this work?	
**Competing Interests**<br><br>Use the instructions below to enter a competing interest statement for this submission. On behalf of all authors, disclose any competing interests that could be perceived to bias this work—acknowledging all financial support and any other relevant financial or non-financial competing interests.<br><br>This statement is required for submission and **will appear in the published article** if the submission is accepted. Please make sure it is accurate and that any funding sources listed in your Funding Information later in the submission form are also declared in your Financial Disclosure statement.<br><br>View published research articles from	The authors have declared that no competing interests exist.

Powered by Editorial Manager® and ProduXion Manager® from Aries Systems Corporation

PLOS ONE for specific examples.<br><br>**NO authors have competing interests**<br><br>Enter: *The authors have declared that no competing interests exist.*<br><br>**Authors with competing interests**<br><br>Enter competing interest details beginning with this statement:<br><br>*I have read the journal's policy and the authors of this manuscript have the following competing interests: [insert competing interests here]*<br><br>* typeset	
**Ethics Statement**<br><br>Enter an ethics statement for this submission. This statement is required if the study involved:<br><br>• Human participants<br>• Human specimens or tissue<br>• Vertebrate animals or cephalopods<br>• Vertebrate embryos or tissues<br>• Field research<br><br>Write "N/A" if the submission does not require an ethics statement.<br><br>General guidance is provided below. Consult the submission guidelines for detailed instructions. **Make sure that all information entered here is included in the Methods section of the manuscript.**	N/A

Powered by Editorial Manager® and ProduXion Manager® from Aries Systems Corporation

**Format for specific study types**<br><br>**Human Subject Research (involving human participants and/or tissue)**<br>• Give the name of the institutional review board or ethics committee that approved the study<br>• Include the approval number and/or a statement indicating approval of this research<br>• Indicate the form of consent obtained (written/oral) or the reason that consent was not obtained (e.g. the data were analyzed anonymously)<br><br>**Animal Research (involving vertebrate animals, embryos or tissues)**<br>• Provide the name of the Institutional Animal Care and Use Committee (IACUC) or other relevant ethics board that reviewed the study protocol, and indicate whether they approved this research or granted a formal waiver of ethical approval<br>• Include an approval number if one was obtained<br>• If the study involved *non-human primates*, add *additional details* about animal welfare and steps taken to ameliorate suffering<br>• If anesthesia, euthanasia, or any kind of animal sacrifice is part of the study, include briefly which substances and/or methods were applied<br><br>**Field Research**<br><br>Include the following details if this study involves the collection of plant, animal, or other materials from a natural setting:<br>• Field permit number<br>• Name of the institution or relevant body that granted permission	
**Data Availability**<br><br>Authors are required to make all data underlying the findings described fully available, without restriction, and from the time of publication. PLOS allows rare exceptions to address legal and ethical concerns. See the PLOS Data Policy and FAQ for detailed information.	Yes - all data are fully available without restriction

Powered by Editorial Manager® and ProduXion Manager® from Aries Systems Corporation

A Data Availability Statement describing where the data can be found is required at submission. Your answers to this question constitute the Data Availability Statement and **will be published in the article**, if accepted.<br><br>**Important:** Stating 'data available on request from the author' is not sufficient. If your data are only available upon request, select 'No' for the first question and explain your exceptional situation in the text box.<br><br>Do the authors confirm that all data underlying the findings described in their manuscript are fully available without restriction?	
**Describe where the data may be found in full sentences. If you are copying our sample text, replace any instances of XXX with the appropriate details.**<br><br>• If the data are **held or will be held in a public repository**, include URLs, accession numbers or DOIs. If this information will only be available after acceptance, indicate this by ticking the box below. For example: *All XXX files are available from the XXX database (accession number(s) XXX, XXX.).*<br>• If the data are all contained **within the manuscript and/or Supporting Information files**, enter the following: *All relevant data are within the manuscript and its Supporting Information files.*<br>• If neither of these applies but you are able to provide **details of access elsewhere**, with or without limitations, please do so. For example:<br><br>*Data cannot be shared publicly because of [XXX]. Data are available from the XXX Institutional Data Access / Ethics Committee (contact via XXX) for researchers who meet the criteria for access to confidential data.*<br><br>*The data underlying the results presented in the study are available from (include the name of the third party*	All relevant data are within the manuscript.

and contact information or URL).<br>• This text is appropriate if the data are owned by a third party and authors do not have permission to share the data.<br><br>* typeset	
Additional data availability information:	

Manuscript

Click here to access/download;Manuscript;Nanoscope Manuscript Final.docx

A comparative analysis of infection rates associated with needle arthroscopy conducted in an operating room versus those performed under sedation, in a nonoperating room setting in canine joints: A retrospective study.

Short title: Outpatient needle arthroscopy shows no increased infection in canine joints

Alessandra Chiaramonte, VMD, CCRP, [1*] Cassio R. A. Ferrigno, DVM, MS, PhD,[1] Darryl L. Millis, DVM, MS, DACVS, CCRP, DACVSMR,[1] Jessica W. Montoya, AAS, LVMT, CCRP, VTS-Surgery, [1]

[1]Department of Surgery, University of Tennessee College of Veterinary Medicine (UT-CVM), Knoxville, Tennessee, United States of America

[*]Corresponding Author

Email: achiaram@utk.edu

Author Contributions

AC, CRAF, DLM contributed to the conception and study design, data acquisition, editorial of the manuscript, and final approval for publication. They are publicly accountable for relevant content relating to this study. JM contributed to the surgical procedure preparation and data acquisition, editorial of the manuscript, and final approval for publication.

Abstract

Objective: To determine infection rates for needle arthroscopy (NA) in client-owned dogs performed in an operating room (OR) versus in a nonoperating room setting (NOR) under heavy sedation. We hypothesized that there would be no difference in infection rate.

Study Design: Retrospective study of 75 dogs (89 individual joints) that underwent needle arthroscopy at one academic institution, the Veterinary Medical Center of the University of Tennessee (UT-CVM), from May 4th, 2022, through December 20th, 2024, were compiled. A formal consent to terms of conditions was signed by each client upon admission to UT-CVM. By signing this consent form, the client authorized the use of the dogs information for clinical research.

Results: Ten cases were excluded due to evidence of infection before arthroscopy for TPLO implant explant (n = 8), bite wound infection (n = 1), or surgical complications not related to the needle arthroscopy (n = 1). Forty-five cases were performed in an operating room under general anesthesia, while thirty cases were performed under heavy sedation in a non-operating room setting. Routine follow-up was performed. Descriptive statistics were reported since the infection rate was 0% for all OR and NOR. There was no difference in infection rate for OR needle arthroscopy or NOR needle arthroscopy.

Conclusion: Needle arthroscopy can be performed on an outpatient basis and without increased risk of a septic joint when performed with appropriate aseptic technique.

Introduction

Septic arthritis following joint arthroscopy is a rare but detrimental post-operative complication. In human medicine, the incidence of infection rates following arthroscopy has been widely reported in multiple joints. The reported incidence of infection following knee arthroscopy in an operating room has been determined to be less than 0.2%, although ranges from 0.009 – 1.1% have been reported.[1,2] Another study observed a 0.26% infection rate for patients undergoing shoulder arthroscopy.[3] Known post-arthroscopic risk factors established in human orthopedic surgery for increased risk of infection include intra-articular corticosteroid injections, smoking, obesity, male gender, revision surgery, increased surgical time, chronic anemia, and malnutrition.[2,3,5]

With the rise of outpatient arthroscopy, a recent human medical study investigated the rate of post-operative infections for knee and shoulder arthroscopy performed in outpatient office stings. From this research, no major post-operative infections were reported.[4] The reported infection rate for conventional arthroscopy in small animal orthopedics ranges from 1-3%.[6,7] A retrospective study following 353 elective conventional stifle arthroscopies, diagnosed only 3 cases with post-operative septic arthritis (0.85%).[7] A study on canine elbow conventional arthroscopy revealed a lower incidence of postoperative septic arthritis at 0.22%.[8]

Although several articles have reported infection rates for conventional arthroscopy, there remains few research on infection rates with needle arthroscopy especially outside the operating

room. Needle arthroscopy has increased in popularity in the veterinary setting over the last five years.[9-12] This is due to the minimally invasive diagnostic accuracy of needle arthroscopy with less equipment and decreased preparation time.[12] Several articles have been published validating the diagnostic accuracy of needle arthroscopy over conventional arthroscopy for medial coronoid disease, medial shoulder instability, and medial meniscal tears in dogs.[9-12] Needle arthroscopy also has reported utility in treating medial coronoid disease via fragment retrieval with favorable outcomes.[13]

Needle arthroscopy offers the ability to perform minimally invasive diagnostics on an outpatient basis. Despite the increase in usage, there have been no studies assessing the incidence of infection when used on an outpatient basis outside of an operating room in dog joints. The objective of this study was to determine the incidence of infection rate for needle arthroscopy performed in a sterile operating room versus under heavy sedation in a nonoperating room with appropriate aseptic technique. We hypothesized that there would be no difference in infection rate for needle arthroscopy performed in either location.

Materials and Methods

Medical records of all dogs undergoing needle arthroscopy at University of Tennessee College of Veterinary Medicine (UT-CVM) were compiled from May 4th, 2022, through December 20th, 2024. An animal research ethics committee was not prospectively approved for this research study given that this was a clinical retrospective study following a surgical treatment. Formal consent to terms of conditions and treatment was signed by each client. The formal consent is as follows:

Veterinary Medical Center of the University of Tennessee

Conditions of Admission/Treatment:

Medical and Surgical Consent: I, the undersigned, do hereby certify that I am the owner (or authorized agent of the owner) of this animal and that I am 18 years of age or older; that I hereby authorized the Veterinary Medical Center of the University of Tennessee, their agents or representatives, to perform the medical or surgical procedures, sedation, anesthesia, x-ray examination, diagnostic procedures, prescribe and administer drugs, or such treatment which the clinical deems necessary. I understand that general anesthesia is routinely used for diagnostic tests. I understand that diagnostic and surgical procedures carry potential risks.

Education: I understand and agree that the University of Tennessee Veterinary Medical Center is involved in educational activities and that faculty, residents, interns, and students will be involved in my animal’s care.

Consent and Release: I hereby authorize the University of Tennessee College of Veterinary medicine and its authorized designees to record, transmit, and store in any format and/or medium (analog, digital, electronic, or other medica now know or later developed) my own and my animal’s name, likeness, images, voice, appearance, data, diagnostics, and/or medical records arising out of and relating to the performance, care, and medical treatment hereunder and, further, hereby grant the institution and its authorized designees the perpetual and irrevocable right to use the same, in whole or part for any commercial or non-commercial purpose, including but not limited to, research, public awareness, media production, publication, or academic application as the institution determines in its sole discussion. **Notwithstanding the foregoing, to the extent I (or my representative) is recorded therein, the University of Tennessee College of Veterinary Medicine will keep such recording strictly confidential per hospital policy and the recording will only be used for purposed of veterinary student education**

unless I (or my representative) provides broader written consent or I am (or my representative is) removed or de-identified in such recording. I expressly release the institution, its officers, employees, agents, and designees from any and all claims known and unknown arising out of or connected with the above granted uses.

The undersigned certifies that he/she has read the foregoing, or has had the foregoing read to him/her, and that he/she understands and fully accepts its terms.

Date: _______ Owner: _____________________________

Date: _______ Authorized Agent: _______________________

This formal consent authorizes UT-CVM to use medical records for retrospective research purposes. No animals were sacrificed or euthanized for this study. Researchers had access to identification of patients involved in this study and were not blinded. Clinical records were obtained for a total of 75 dogs undergoing needle arthroscopy for 89 individual joints. Additional data attained from the medical records included signalment, age at the time of surgery, gender, breed, dog size, laterality of clinical signs, and any pre-existing conditions that may affect infection rates such as endocrinopathies, obesity, and concurrent infections. Dog size was stratified according to weight. Small dogs were less than 10 kg, medium dogs weighed 11-20 kg, and large dogs weighed greater than 21 kg. Intra-operative data included any post-arthroscopic complications, including incisional site infection, positive culture results indicative of septic arthritis, conversion to an open procedure, need for a second arthroscopic procedure, or worsening lameness/unresolved lameness because of osteoarthritis, were collected.

All needle arthroscopy procedures were performed with a sterilized 1.9 mm Arthrex Nanoscope Camera and Visualization System (Arthrex Vet Systems, Naples, Florida, United

States) (Figure 1). The location for the needle arthroscopy procedure, OR under general anesthesia versus NOR outpatient under heavy sedation, was determined based on the diagnosis. If a patient required surgical intervention along with arthroscopy, both procedures were performed in the OR. If the physical and orthopedic examination did not indicate surgical intervention or surgical intervention was to be performed later, needle arthroscopy was performed on an outpatient basis (NOR) (Figure 2).

All surgical procedures were performed by faculty surgeons or a surgical resident under direct supervision. An anesthetic protocol was tailored to each individual patient based on anesthesiologist's or clinician's preference. The joints of patients undergoing an additional procedure were prepped with a standard protocol of 4% chlorhexidine scrub (BD E-Z Scrub impregnated with 3% chloroxylenol, Becton Dickinson and Company, Franklin Lakes, New Jersey, United States) followed by a dilute 2% chlorhexidine scrub (Chloradine Scrub 2%, Aspen Veterinary Resources, Liberty, Missouri, United States) after clipping the area. The surgical site was then sterilely prepared with ChloraPrep (2% chlorhexidine gluconate & 70% isopropyl alcohol, Becton Dickinson and Company, Franklin Lakes, New Jersey, United States) for 3 minutes prior to initial skin incision in the operating room. Patients undergoing arthroscopy in the OR also received a prophylactic dose of cefazolin (22 mg/kg, 100 mg/mL, 1gm/vial, NovaPlus, Hikma Pharmaceuticals, Berkeley Heights, New Jersey, United States) intravenously.

An alternating dilute 2% chlorhexidine and 70% alcohol scrub was performed on joints undergoing needle arthroscopy outside of the operating room (NOR). The aseptic solution was allowed to dry prior to the initial skin incision. Outpatient needle arthroscopy patients did not receive prophylactic antibiotics.

Exclusion criteria

Ten cases exhibited symptoms concerning infection resulting from a previous procedure; thus, they were excluded from the data set. Of these ten cases, eight had evidence of infection prior to needle arthroscopy and were scheduled for a TPLO implant removal. These cases were explanted with positive culture growth. One case was infected due to a chronic bite wound. The last case was excluded for a canine unicompartmental elbow (CUE) implant associated infection.

Follow-up

Follow-up was determined by the case, according to the diagnosis and procedure. Recheck examinations were scheduled based on the surgical procedure completed following the needle arthroscopy diagnosis.

Statistical Analysis

Descriptive statistics such as signalment were reported. The mean, median, standard deviation, and range were calculated for age and weight. The infection rates for needle arthroscopy in the OR and NOR were both 0%, precluding further statistical analysis.

Results

Needle arthroscopy was performed in 75 cases, including 89 individual joints. Forty-five needle arthroscopies were performed under general anesthesia in an OR, while thirty cases were performed under heavy sedation in NOR on an outpatient basis (Tables 1 & 2). Ten patients had more than one joint arthroscopically examined under the same anesthetic or sedative event.

Table 1: Summary of results from needle arthroscopy cases performed in the operating room (OR).

Intraoperative Needle Arthroscopy Cases	45 cases
Average Age	5.6 years
Average Weight	27.4 kg

Follow-Up	Mean 57.8 (± 0.87) days<br>Range 0 – 365 days	
Sex		
Intact Male	8	
Castrated Male	14	
Intact Female	3	
Spayed Female	20	
Joint	Number	Diagnosis
Carpus	Left = 0	Incomplete ossification of radial carpal bone
	Right =1	
Elbow	Left = 3	Fragmented medial coronoids (FMCPs), elbow dysplasia
	Right = 4	
Shoulder	Left = 4	Medial glenohumeral ligament tear, supraspinatus tendon tear, biceps tenosynovitis
	Right =6	
Tarsus	Left = 1	Osteochondritis dissecans (OCD)
	Right = 0	
Stifle	Left = 19	Cranial cruciate ligament rupture (CCLR), medial meniscal tear, osteoarthritis
	Right =16	
Hip	Left = 2	Hip dysplasia, osteoarthritis

Table 2: Summary of results from needle arthroscopy cases performed outpatient in a nonoperating room environment (NOR).

Outpatient, Nonoperating Room Needle Arthroscopy Cases	30 cases	
Average Age	5.9 years	
Average Weight	27.5 kg	
Follow-Up	Mean 42.6 (± 1.00) days<br>Range 0 - 425 days	
Sex		
Intact Male	8	
Castrated Male	10	
Intact Female	2	
Spayed Female	10	
Joint	Number	Diagnosis
Carpus	Left = 0	
	Right = 0	
Elbow	Left = 1	Fragmented medial coronoids (FMCPs), elbow dysplasia
	Right = 1	
Shoulder	Left = 3	Osteoarthritis, cartilage erosion of humeral head, supraspinatus tendon tear, biceps tenosynovitis
	Right =6	
Tarsus	Left = 0	

	Right = 0	
Stifle	Left = 5	Cranial cruciate ligament rupture (CCLR), medial meniscal tear, osteoarthritis, patellar desmitis
	Right = 11	
Hip	Left = 3	Osteoarthritis, hip dysplasia

The mean age of patients at the time of the procedure was 5.6 years for OR and 5.9 years for NOR. OR cases were as follows: eight dogs intact males, fourteen were castrated males, three intact females, and twenty spayed females. NOR cases were eight intact males, ten castrated males, two intact females, and ten spayed females. Fifty-five dogs were categorized as large (≥ 20kg), fourteen medium (≤ 10 kg - < 20kg), and six small (10 kg). The average weight was 27.4 kg in OR and 27.5 in NOR. Large breed dogs were the most represented group in this study population. It is reasonable to postulate that this is due to higher incidence of orthopedic conditions able to be treated in a minimally invasive manner in this population.[8-13] Eight cases had repeat arthroscopies for the same previously diagnosed issue: four OR, two NOR, and two initially performed NOR then moved into the OR based on diagnosis. Ten patients had more than one joint arthroscopically examined under the same anesthetic or sedative event.

A total of 89 individual joints underwent needle arthroscopy including one right carpus, four left cubital joints, five right cubital joints, seven left glenohumeral joints, twelve right glenohumeral joints, one left tarsus, twenty-four left stifles, twenty-six right stifles, five left coxofemora, and four right coxofemora joints (Table 1 & 2). Diagnoses included medial coronoid disease, biceps/supraspinatus tendinopathy, complete or partial cranial cruciate ligament ruptures, medial meniscal tears, patellar tendon desmitis, or coxofemoral osteoarthritis. Nine patients underwent a second-look stifle arthroscopy after a TPLO to assess for possible late meniscal tear or other post-operative complications.

Intraoperative complications were recorded, including conversion to an open tarsal and stifle arthrotomy procedure due to difficulty with fragment removal in two cases. No post-operative complications were associated with the needle arthroscopy. Forty-five of the cases were performed under general anesthesia in an OR while thirty cases were performed under heavy sedation in NOR on an outpatient basis. Out of the 85 total cases, no infections were appreciated in either group.

The mean time for follow-up of cases was 57.8 (± 0.87) days for OR (range 0 – 365 days) and 42.6 (± 1.00) days for NOR (range 0 -425 days) post-arthroscopy. During this time, no cases were noted to have concerns of septic arthritis based on physical examination, orthopedic examination, and/or radiographic evaluation. Nine cases were lost to follow-up, and seventeen cases were rechecked with their primary care veterinarian to assess incision healing or scheduled rechecks at UT-CVM Orthopedics Service only if lameness recurred.

Discussion

Our results led us to accept our hypothesis that there was no difference in infection rate for needle arthroscopy performed in an OR versus NOR (0%).[2,3,5,6] To the authors knowledge, this is the first study that documents outpatient needle arthroscopy performed outside of an operating room for veterinary patients. The results of this study lead us to conclude that outpatient needle arthroscopy in an uncontrolled, nonoperating room environment did not pose an increased risk for post-arthroscopic joint infection.

In human medical and veterinary patients, septic joint arthritis is most commonly secondary to hematogenous spread, however, post-operative infections are possible. It can have detrimental effects including cartilage damage, permanent loss of limb function, or lead to limb

amputation when not addressed promptly and aggressively.[6,14] Thus, given the growing popularity of needle arthroscopy, the absence of statistically significant increased risk of infections when needle arthroscopy is performed in an uncontrolled, nonoperating room is vital insight. The results of this paper lead us to conclude that needle arthroscopy is a viable cage side diagnostic test on an outpatient basis in sedated patients without an increased risk of infection.

A major limitation to this retrospective study is that bacterial cultures were not performed routinely. Cultures were only performed if there was concern or suspicion for septic joint effusion based on clinical signs, radiography, and cytology. Additional limitations of this study include its retrospective nature and the small sample size in each group. In the future, a larger prospective clinical trial should be performed with preoperative and postoperative cultures. A total of 514 needle arthroscopies (257 in the operating room and 257 outpatient) would be required to achieve a strong power study of 0.8 and alpha of 0.05 (power study performed using GraphPad Prism 9.4.1, Dotmatics, 2025).

In conclusion, there is no difference in infection rate for needle arthroscopy performed inside or outside an operating room setting. Septic bacterial arthritis continues to be very uncommon post-arthroscopy complication in dog. Needle arthroscopy can be performed on an outpatient basis and without increased risk of a septic joint when performed with appropriate aseptic technique.

References

1. Marmor S, Farman T, Lortat-Jacob A. Joint infection after knee arthroscopy: medicolegal aspects. Orthop Traumatol Surg Res. 2009;95(4):278-83.

2. Balato G, Di Donato SL, Ascione T, D'Addona A, Smeraglia F, Di Vico G, et al. Knee Septic Arthritis after Arthroscopy: Incidence, Risk Factors, Functional Outcome, and Infection Eradication Rate. Joints. 2017;5(2):107-13.
3. Cancienne JM, Brockmeier SF, Carson EW, Werner BC. Risk Factors for Infection After Shoulder Arthroscopy in a Large Medicare Population. Am J Sports Med. 2018;46(4):809-14.
4. McMillan S, Chhabra A, Hassebrock JD, Ford E, Amin NH. Risks and Complications Associated With Intra-articular Arthroscopy of the Knee and Shoulder in an Office Setting. Orthop J Sports Med. 2019;7(9):2325967119869846.
5. Forsythe B, Berlinberg EJ, Forlenza EM, Oeding JF, Patel HH, Mascarenhas R. Corticosteroid injections 2 months before arthroscopic meniscectomy increases the rate of postoperative infections requiring surgical irrigation and debridement. Knee Surg Sports Traumatol Arthrosc. 2022;30(11):3796-804.
6. Clements DN, Owen MR, Mosley JR, Carmichael S, Taylor DJ, Bennett D. Retrospective study of bacterial infective arthritis in 31 dogs. J Small Anim Pract. 2005;46(4):171-6.
7. Ridge PA. A retrospective study of the rate of postoperative septic arthritis following 353 elective arthroscopies. J Small Anim Pract. 2011;52(4):200-2.
8. Perry KL, Li L. A retrospective study of the short-term complication rate following 750 elective elbow arthroscopies. Vet Comp Orthop Traumatol. 2014;27(1):68-73.
9. Hersh-Boyle RA, Chou PY, Kapatkin AS, Spriet M, Filliquist B, Garcia TC, et al. Comparison of needle arthroscopy, traditional arthroscopy, and computed tomography for the evaluation of medial coronoid disease in the canine elbow. Vet Surg. 2021;50 Suppl 1:O116-o27.

10. Garnier P, Decambron A, Manassero M, Viateau V. Needle arthroscopy for exploration of the elbow joint: a case series of six dogs with preliminary cadaveric study. N Z Vet J. 2022;70(5):287-96.

11. von Pfeil DJF, Megliola S, Horstman C, Tan D, Glassman M. Comparison of classic and needle arthroscopy to diagnose canine medial shoulder instability: 31 cases. Can Vet J. 2021;62(5):461-8.

12. Evers JS, Kim SE, Johnson MD, Lazarus MA. Accuracy of needle arthroscopy for the diagnosis of medial meniscal tears in dogs with cranial cruciate ligament rupture. Vet Surg. 2023;52(6):820-6.

13. Garnier P, Dekerle B, Vial J, Maurice E, Manassero M, Viateau V. Evaluation of a small-bore needle arthroscope for diagnosis and treatment of medial coronoid disease in dogs: a pilot study with short-term assessment. N Z Vet J. 2023;71(3):152-8.

14. Wind WM, McGrath BE, Mindell ER. Infection following knee arthroscopy. Arthroscopy. 2001;17(8):878-83.

Figure 1 Arthrex Nanoscope Camera and Visualization System

Click here to access/download;Figure;Figure 1 - Arthrex Nanoscope Camera and Visualization System.png

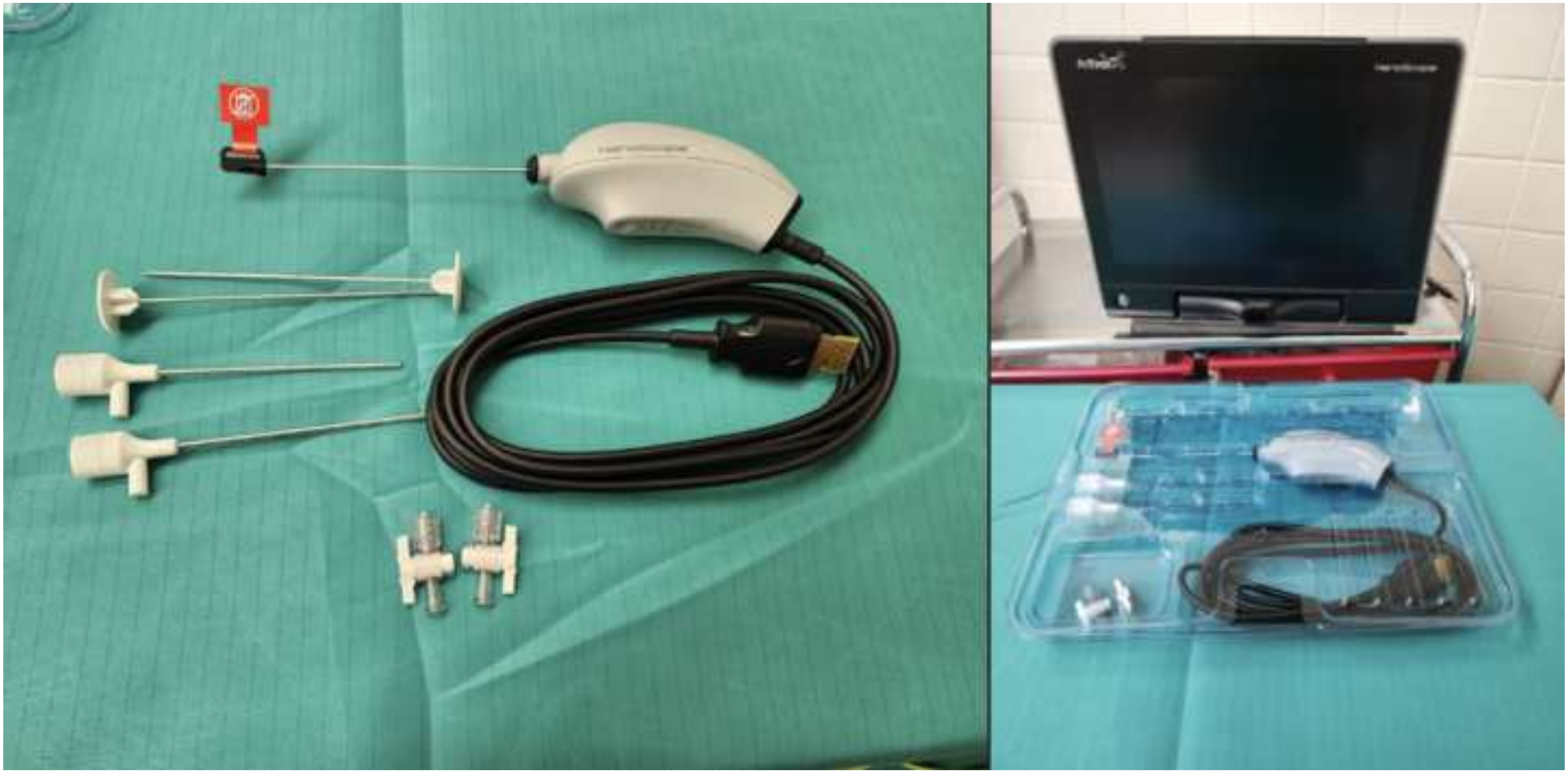

Figure 2 Needle arthroscopic set up for outpatient, nonoperating room setting

Click here to access/download;Figure;Figure 2 - Needle arthroscopic set up for outpatient NOR.png

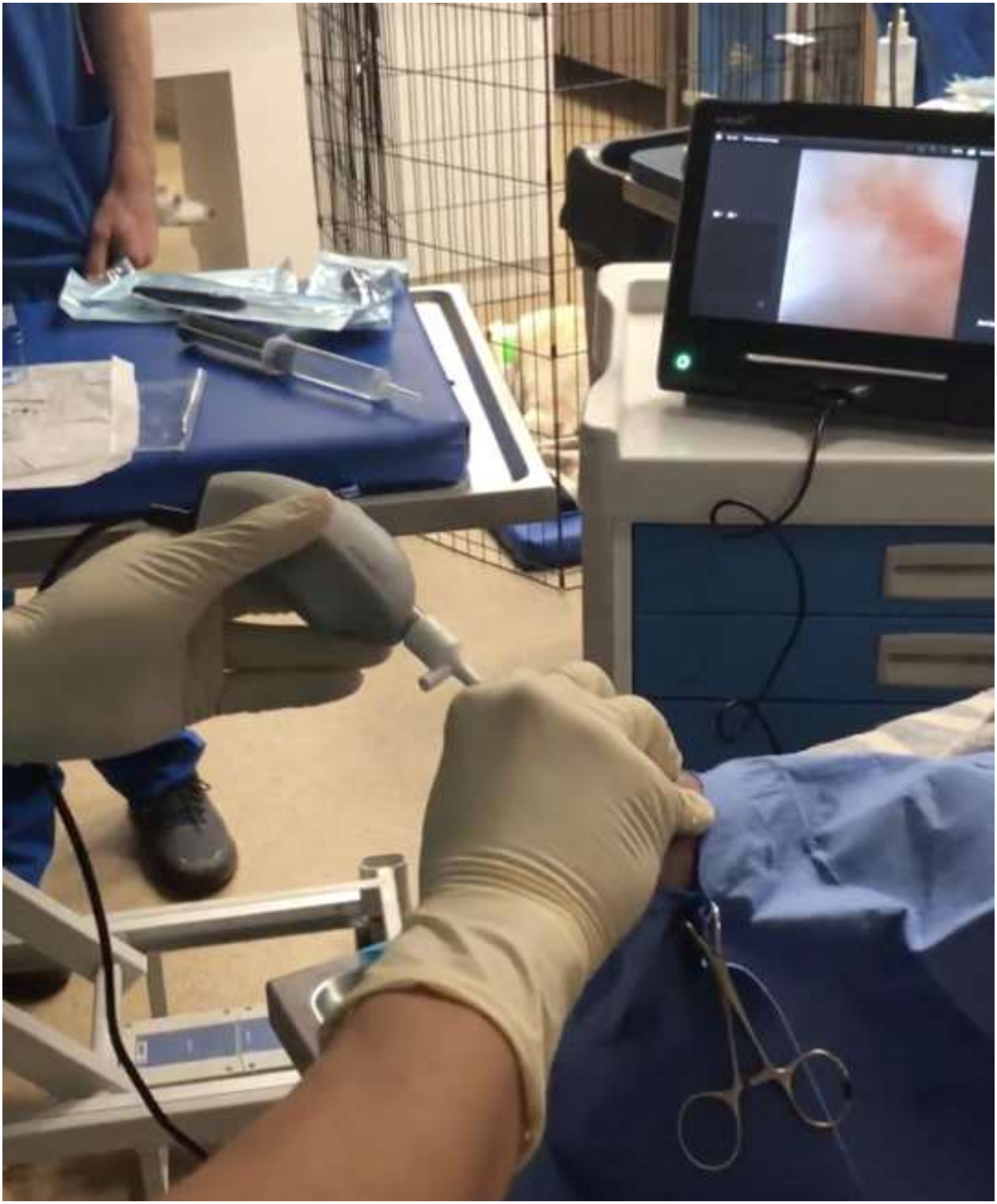

Supplement: Attachment — Submitted filename: PONE-D-25-49231 Needlescope review.pdf [file pone.0343878.s001.pdf]
