# Supplementary material for: Observed joint infection incidence following needle arthroscopy performed in operating and nonoperating room environments in client-owned dogs: A retrospective cohort study
Source: PLoS One. 2026 Feb 25;21(2):e0343878. doi: 10.1371/journal.pone.0343878 (PMC12935261; doi:10.1371/journal.pone.0343878)
Supplement: Attachment — Submitted filename: Response to Reviewers.docx [file pone.0343878.s002.docx]

Dear Plos One Reviewers,

Thank you for your thorough review and comments of our manuscript titled “A comparative analysis of the infection rates associated with needle arthroscopy conducted in an operating room versus those performed under sedation in a nonoperating room setting in canine joints: a retrospective study” retitled Observed Joint Infection Incidence Following Needle Arthroscopy Performed in Operating and Nonoperating Room Environments in Client-Owned Dogs: A Retrospective Cohort Study. The authors appreciate your valuable insight which has allowed us to improve the quality of our original manuscript. We have addressed the specific comments raised in detail below.

-----------------------------------------------------------------------------------------------------------------

Reviewer #1: Mustafa Akkaya

1. Comment: Title consider simplifying wording and avoid long phrasing
   a. Response: We agree with your suggestion. The title of our manuscript has been revised and shortened to more explicitly relay the purpose and results of this research.
2. Comment: Abstract. State follow-up duration more explicitly. Ass a sentence clarifying no inferential statistics were performed due to zero infection event rate. Mention study design as a retrospective.
   a. Response: These have all been addressed in the revised manuscript and clearly stated.
3. Comment: Introduction. Clarify why infection rates may theoretically differ between OR and outpatient sedated settings. Strengthen motivation: emphasize the clinical relevance of avoiding general anesthesia and using minimally invasive diagnostic options.
   a. Response: This has been further clarified and addressed in lines 61-66 in the revised manuscript.
4. Comment: Methods.
   a. Clearly state infection definition.
      i. Response: Definition of septic arthritis or joint associated infection included lines 95-98
   b. Thoroughly describe outpatient procedural environment.
      i. Response: Lines 105-108
   c. Specify how follow-up was conducted. Indicate how many cases were lost to follow-up.
      i. Response: Lines 139-147
   d. Rationale for antibiotic protocol should be briefly justified.
      i. Response: Please see explanation for rationale in lines 118-131. Included are four human references that this continues to be a controversial topic.
5. Comment: Results. Clarify follow-up duration. Specify how many cases had repeat arthroscopy and whether this was related to unresolved pathology versus potential infection concerns. If possible include a small table summarizing follow-up type.
   a. Response: Duration has been clarified in the tables provided. Follow-up was also more clearly explained in lines 1390147. Further clarification was included for the repeat arthroscopy procedures lines 174-177. Only one arthroscopic case was concerning for infection and repeated cultures of synovial fluid showed no growth.
6. Comment: Discussion. Expand on clinical implications of being able to safely perform NA outside the OR. Limitations should be more prominently discussed.
   a. Response: We have included an expansion of why it is important to be able to perform NA outside the OR due to decreased cost to client without general anesthesia, bench side diagnostic test that offers quick results, and limits invasive techniques. We outright acknowledge the possibility of type II error and clarify that the absence of observed infections does not equate to proof of zero risk.
7. Comment: Conclusion. Avoid implying equivalence without statistical testing. Use cautious phrasing.
   a. Response: We agree with your comments. Our conclusion emphasizes that no infections were observed rather than implying a definitive absence of infection risk. It now clearly states this study as preliminary research that justifies a larger prospective clinical trial in the future.

---------------------------------------------------------------------------------------------------------------------

Reviewer #2:

Dear Reviewer #2,

Thank you for your evaluation of our manuscript and insightful critiques. We appreciate your points regarding the small study population and your concern about meeting our objective. We agree with the reviewer that provided with the low expected incidence of post-arthroscopic infections and limited sample size, our study is not powered to determine true infection rate or detect rare adverse events. We have edited this distinction in our manuscript accordingly by emphasizing the observational assessment of post-arthroscopic infections and changed the language throughout the manuscript to avoid implying definitive infection rate was established. We did change our objective to be two-fold – one, to report the use of outpatient needle arthroscopy since it has not been published in veterinary medicine as an outpatient modality, and to compare our observed infections between nonoperating and operating room environment.

Materials & Methods: Per your recommendation, the “Conditions of Admission/Treatment” aka consent form was moved to supplemental documentation.

Line 172-173: Further addressed that the two cases that were moved into the OR underwent general anesthesia for a longer surgical procedure and thus were included in the OR group.

Line 177-179: If a patient had multiple joints scoped, they were counted individually. Yes, this would have been interesting if any of the joints became infected!

To your point on antibiotics – Our other reviewer also expressed these concerns. Please refer to lines 118-131.

Discussion: We added a dedicated paragraph to our discussion section that addresses expected infection incidence based on published literature and explains why our sample size limits detection of rare events. The power analysis we felt conceptualized the estimate for future prospective work. We outright acknowledge the possibility of type II error and clarify that the absence of observed infections does not equate to proof of zero risk.

Conclusion: We agree and now emphasize that no infections were observed rather than implying a definitive absence of infection risk. The concluding paragraph now clearly states this study as preliminary research that supports feasibility and justifies a larger prospective clinical trial.

---------------------------------------------------------------------------------------------------------------------

We are grateful for the insightful critiques, which have substantially improved the clarity, accuracy, and scientific rigor of our manuscript. We believe that this revised version now appropriately displays our findings, acknowledges our limitations, and contributes meaningful preliminary data regarding outpatient needle arthroscopy in canines.

We hope that these revisions meet your expectations and enhance the clarity of our research. Thank you once again for your constructive feedback and time.

Sincerely,

Alessandra Chiaramonte, VMD

On behalf of all authors

University of Tennessee College of Veterinary Medicine

Small Animal Surgery Department

achiaram@utk.edu
